# Susceptibility to Adrenal Crisis Is Associated With Differences in Cortisol Excretion in Patients With Secondary Adrenal Insufficiency

**DOI:** 10.3389/fendo.2022.849188

**Published:** 2022-04-20

**Authors:** Annet Vulto, Martijn van Faassen, Michiel N. Kerstens, André P. van Beek

**Affiliations:** ^1^ Department of Endocrinology, University Medical Center Groningen, University of Groningen, Groningen, Netherlands; ^2^ Department of Laboratory Medicine, University Medical Center Groningen, University of Groningen, Groningen, Netherlands

**Keywords:** biomarkers, adrenal crisis, pharmacokinetics, cortisol, hydrocortisone, kynurenine, susceptibility to adrenal crisis

## Abstract

**Objective:**

To compare cortisol pharmacokinetics and pharmacodynamics mapped through several glucocorticoid sensitive pathways in patients on hydrocortisone substitution with or without an adrenal crisis.

**Design:**

A *post-hoc* analysis of a previously conducted randomized controlled trial in patients with secondary adrenal insufficiency examining the effects of 2 weight-adjusted hydrocortisone doses.

**Methods:**

Comparisons were primarily made on a hydrocortisone dose of 0.2-0.3 mg/kg/day for plasma cortisol and cortisone, 24-hour urinary steroid profile, the glucocorticoid sensitive tryptophan-kynurenine pathway, the renin-angiotensin-aldosterone system and aspects of quality of life. Variables of interest were also analyzed on the hydrocortisone dose of 0.4-0.6 mg/kg/day.

**Results:**

Out of 52 patients, 9 (17%) experienced at least one adrenal crisis (AC+ group) and 43 did not develop an adrenal crisis (AC- group) during an observation period of 10 years. 24-hour urinary excretion of cortisol and cortisone were lower in the AC+ group (0.05 [IQR 0.03; 0.05] vs. 0.09 [0.05; 0.12] µmol/24h, P=0.01and 0.13 [0.10; 0.23] vs. 0.24 [0.19; 0.38] µmol/24h, P=0.04, respectively). No differences in pharmacokinetics of cortisol were observed. Kynurenine concentrations were higher in the AC+ group (2.64 [2.43; 3.28] vs. 2.23 [1.82; 2.38] µmol/L, P=0.03) as was general fatigue (Z-scores 1.02 [-0.11; 1.42] vs. -0.16 [- 0.80; 0.28], P=0.04). On the higher hydrocortisone dose urinary excretion of cortisol and cortisone was still significantly lower between the AC- and AC + group. The differences in glucocorticoid sensitive variables disappeared.

**Conclusion:**

Patients susceptible to an adrenal crisis demonstrated differences in cortisol and cortisone excretion as well as in pharmacodynamics when compared to patients who did not experience an adrenal crisis, suggesting a biological predisposition in certain patients for the development of an adrenal crisis.

## 1 Introduction

Adrenal insufficiency (AI) is characterized by loss of endogenous cortisol production. The pathophysiology is designated according to the level at which the hypothalamus-pituitary-adrenal gland (HPA)-axis is affected, i.e. tertiary (TAI), secondary (SAI) or primary insufficiency (PAI) for insufficiency at the level of the hypothalamus (i.e., CRH deficiency), pituitary gland (i.e., ACTH deficiency) or adrenal cortex, respectively (1).. Patients suffering from AI are at risk to develop an adrenal crisis (AC).

AC is an acute life- threatening medical situation which is caused by an absolute or relative cortisol deficiency (2, 3). With an incidence of approximately 5-10 per 100 patient-years it is not an infrequent medical emergency (2, 4–7). The risk of AC is higher in PAI than in SAI, probably because of some residual cortisol secretion in some patients with SAI and the lack of mineralocortioid secretion in PAI (6). Patients with TAI, which is most commonly due to glucocorticoid treatment, are at low risk to develop an AC, but precise data are not available (8). AC is a potentially life-threatening clinical condition and an important cause of death in patients with AI, with a mortality rate of 0.5 per 100 patient-years (9).

Previous studies have shown that patients with adrenal insufficiency have a 2-fold greater mortality rate compared to the background population. Main causes of death were cancer, cardiovascular and infectious disease, the latter being possibly driven by inadequate glucocorticoid exposure, especially at times of intercurrent illness (10). With only limited studies available, the mortality rate from AC is estimated to be 0.3-0.5/100 patient years (2, 5, 6, 11, 12).

Little is known which factors or vulnerabilities contribute to the development of an adrenal crisis. It is likely that the occurrence of an AC revolves around three distinctive pillars. Firstly, the type and severity of the event (e.g. infection, surgery, injury or mental stress) in relation to the patient’s vulnerability as determined by general characteristics such as age and comorbidities (3, 6, 13). It has been shown that the risk of an AC was associated with older age and (bacterial) infections, especially gastro-intestinal infections in addition to comorbidities such as diabetes and asthma (2, 5, 6, 14). Furthermore, the etiology of the hypoadrenalism is of relevance, considering that patients with SAI experience less AC compared to patients with PAI. Secondly, patient education on sick day rules is widely believed to be of importance, although this is not without controversy because in a study by Hahner and colleagues perceived patient education on crisis prevention did not correlate with the frequency of an AC or emergency glucocorticoid administration (6). Nevertheless, patient information is widely implemented, and extensive education is nowadays recommended both by professional and patient organizations. Lastly, a biological predisposition related to the hypothalamus –pituitary-adrenal (HPA) axis may be of importance. This predisposition could at least in part explain why 20–44% of patients with adrenal insufficiency develop one or more episodes of AC during their life, while the remainder of patients will never experience such a crisis (4, 9, 15, 16). This could be related to variability in hydrocortisone pharmacokinetics or pharmacodynamics or potential residual (endogenous) cortisol production. Previously, we showed that half of the patients with secondary adrenal insufficiency (SAI) had detectable amounts of 11-deoxycortisol, a marker of residual adrenal cortisol production (17). In addition, slow and fast metabolizers were identified, with a 10-fold difference in plasma cortisol exposure between groups who received a similar weight adjusted hydrocortisone dose (18). However, because cortisol pharmacokinetics may not accurately reflect the effects of glucocorticoid receptor activation, it is of interest to investigate cortisol pharmacodynamics as well. This can be done, for instance, by evaluating the effect of hydrocortisone treatment on certain glucocorticoid sensitive pathways such as the renin-angiotensin-aldosterone system and the tryptophan-kynurenine pathway.

In order to gain more insight into the potential biological determinants of an adrenal crisis, we conducted an exploratory study comparing several aspects of cortisol pharmacokinetics and pharmacodynamics in patients with and without a history of an adrenal crisis.

## 2 Materials and Methods

### 2.1 Study Population

We performed a *post-hoc* analysis of a previously conducted randomized, double-blind crossover study conducted at the University Medical Center Groningen, The Netherlands (ClinicalTrials.gov NCT01546922). An extensive description of the study design is available elsewhere (18). In short, patients with secondary adrenal insufficiency were selected from the outpatient clinic of the University Medical Centre Groningen. All patients suffered from SAI as a consequence of (treatment of) underlying pituitary disease. Inclusion criteria were subjects aged between 18 and 70 years, on stable hydrocortisone substitution or if applicable additional hormone substitutions for at least 6 months. Exclusion criteria were use of drugs interacting with hydrocortisone, diabetes mellitus, and any other major medical condition. The initial cohort of patients participating in the RCT comprised of 60 patients. For this exploratory analysis laboratory measurements were available in a total number of 52 patients. Comorbidities including medication were documented.

### 2.2 Protocol

Patients on cortisone acetate were converted to hydrocortisone 4 weeks prior to baseline measurements. After this run-in phase of 4 weeks, patients randomly received a lower (0.2-0.3 mg hydrocortisone/kg/day) followed by a higher (0.4-0.6 mg/kg/day) hydrocortisone dose or vice versa, both for 10 weeks. Hydrocortisone was given in three divided doses before meals (breakfast, lunch and dinner). In case of intercurrent illness or fever patients were advised to double or triple their HC dose according to a fixed protocol for a maximum of seven days.

On the visit days, patients were instructed to take their morning dose of hydrocortisone at 0700 h. At 0800 h they attended to the hospital and fasting blood samples were drawn in sitting position after a short period of rest for the measurement of plasma total cortisol and plasma free cortisol, which was repeated approximately five hours later. These samples were used for pharmacokinetic analysis. One day before the hospital visit, they collected 24h urine used for steroid profiling and determination of cortisol and cortisone excretion.

Health-related quality of life measures were collected by daily diaries. We used the patient health questionnaire -15 (PHQ-15), general anxiety disorder 7 (GAD-7) and patient health questionnaire 9 (PHQ-9) and after each study period patients completed the Hospital Anxiety and Depression Scale (HADS), Rand 36-Item Health Survey (RAND-36) and Multidimensional Fatigue Inventory (MFI 20).

The study protocol was approved by the University Medical Center Groningen institutional review board. All patients provided written informed consent before participating in the study.

### 2.3 Outcome Parameters

We defined an adrenal crisis as an acute deterioration of a patient’s general health, for which visit to the hospital was deemed necessary and with acute improvement after intravenous administration of glucocorticoids (2, 3). We included retrospectively every AC occurring during the observation period from 2009 to 2019. For every AC, we recorded prior dose adjustments (doubling or tripling the dosage hydrocortisone or cortisone acetate), whether or not hydrocortisone sodium succinate (Solu-Cortef Act-O-Vial^®^) was injected intramuscularly at home, in-hospital treatment (hydrocortisone dosage, admittance to the general ward or intensive care, length of hospital stay) and likely cause or precipitating factors. Furthermore, plasma concentrations of sodium and potassium were recorded as well as blood pressure and heart rate at the time of admission.

### 2.4 Laboratory Measurements

A detailed description of the urinary steroid profiling analysis in urine has been described elsewhere (18, 19). In short, urine samples were enzymatically hydrolysed, stable isotope labeled internal standards 11-keto-etiocholanolone -d5, dehydroepiandrosterone -d6, pregnenolone-d4 and tetrahydrocortisone-d5 were added and unconjugated steroids were extracted from urine by using solid phase extraction. The eluate was evaporated and a two-step derivatization was performed before analysis by gas chromatography in combination with tandem-mass spectrometry.

Plasma steroids and serum tryptophan, kynurenine, and 3-hydroxykynurenine concentrations were analyzed by a validated automated online solid-phase extraction–liquid chromatographic–tandem mass spectrometric method with deuterated internal standards, as previously described (20, 21). Plasma equilibrium dialysis for free cortisol was performed using a 10k cellulose membrane, to create CBG free plasma. The procedure was further performed as described by Fiers et al. (22). LC-MS/MS was applied for measurement of free cortisol as well as for measurement of 11-deoxycortisol, corticosterone and 11-deoxycorticosterone (17, 19).

Plasma renin concentration was measured with an immunoradiometric renin assay (Renin III Generation^®^; Cisbio). Aldosterone in serum was measured by LCMS/MS, essentially as described by Van der Gugten et al, but using additional online solid-phase extraction in combination with LC-MS/MS analysis (19, 23). The method was validated by evaluating imprecision, limit of quantification (LOQ), linearity, carryover, recovery, and ion suppression. Intrassay imprecision (n= 20 at one day) was < 5.6% (at 110, 288, and 1259 pmol/L), whereas interassay imprecision (n = 20 different days) was < 6% (at 110, 292, and 1260 pmol/L). Recovery was evaluated using by spiking three different samples with three increasing concentrations of aldosterone (92, 461 and 925 pmol/L). Recovery was found to be within 92 – 112%. LOQ was 19 pmol/L.

### 2.5 Pharmacokinetic Parameters

Pharmacokinetic parameters were calculated as described by Werumeus Buning et al. (18). In short, one-compartment and two-compartment population models for plasma total cortisol was calculated using the Kinpop Module of MwPharm version 3.81. The one-compartment model showed the best fit and was used for further analyses. Total body clearance (CL), volume of distribution (V_d_), elimination half‐life (T_½_) and area under the curve in one hour (AUC_1_) and 24 hours (AUC_24h_) were calculated.

### 2.6 Glucocorticoid Sensitivity Altering Glucocorticoid Receptor Polymorphisms

We genotyped all patients for GR hypersensitive (1/2 copies BclI and/or N363S) and GR resistant (1/2 copies ER22/23EK and/or 9β) variants (24).

### 2.7 Statistics

Data were analysed using SPSS version 23.0. Normality of data was assessed by Q-Q plots. Normally distributed data is presented as mean (SD), non-normally distributed data is presented as median (IQR). Analysis of data was primarily performed when using the lower dose of hydrocortisone (i.e. 0.2-0.3 mg/kg/day) as this was considered to better reflect a state of relative hypocortisolism. A two-sided P value <0.05 was considered significant. Because of the exploratory character of this study all P < 0.1 were deemed of potential interest. These variables of potential interest were also tested in the higher dose condition (0.4-0.6 mg/kg/day). Differences in baseline characteristics, urinary cortisol, cortisone and steroid profiles, pharmacokinetic parameters and other laboratory measurements in patients with and without an AC were assessed by Mann-Withney U tests or χ2 tests, where appropriate.

## 3 Results

### 3.1 Baseline Characteristics

In total, 52 patients were included in this study. During the timeframe 2009-2019, 9 (17%) of these patients suffered from at least one AC. This group did not differ from the group without any adrenal crisis in age or sex, nor in educational level, age at diagnosis, duration of substitution therapy, BMI or type and dosage of glucocorticoid treatment (**Table 1**). Out of 52, 13 (25%) patients were treated with medication that interfered with the renin-angiotensin-aldosterone system (**Table 1**).

**Table 1:** Clinical characteristics of patients with secondary adrenal insufficiency with or without a history of an adrenal crisis.

		Total patient group (n = 52)	Adrenal Crisis - (n = 43)	Adrenal Crisis + (n = 9)	P-value
Age (years), median [IQR]		54 [43; 61]	55 [45; 61]	54 [23; 64]	0.70
Sex (males/females), n		30/22	25/18	5/4	0.91
Educational level (1/2/3/4/5/6/7) ♦		0/2/1/7/24/16/2	0/2/1/6/19/13/2	0/0/0/1/5/3/0	0.85
Age at diagnosis (years), median [IQR]		33 [21; 46]	34 [22;47]	22 [17; 52]	0.67
Duration of adrenal insufficiency (years)		18.2 [11.4; 27.9]	18.4 [11.5; 32.0]	14.6 [8.0; 20.5]	0.16
Childhood onset/Adult onset of SAI, n		7/45	5/38	2/7	0.59
Body weight (kg), median [IQR]		85.7 [72.6; 93.7]	86 [72; 94]	85 [70; 94]	0.63
BMI (kg/m²), median [IQR]		26.8 [24.5; 30.1]	26.7 [24.5; 29.9]	27.1 [24.2; 31.9]	0.65
Systolic blood pressure (mmHg) median [IQR]		135 [126; 147]	136 [127, 147]	126 [117; 156]	0.54
Diastolic blood pressure (mmHg), median[IQR]		78 [70; 86]	78 [70; 85]	79 [67; 93]	0.57
eGFR (CKD-EPI, ml/min/1.73m^2^), median [IQR]		82 [69; 95]	81 [69; 95]	88 [67; 99]	0.57
No. of hormonal replacement (1/2/3/4/5)		3/10/23/13/3	3/8/20/10/2	0/2/3/3/1	0.73
	Thyroid hormone (yes/no)	48/4	39/4	9/0	1.0
	Growth hormone (yes/no)	24/28	19/24	5/4	0.72
	Sex hormone (yes/no)	29/23	24/19	5/4	1.0
	Desmopressin (yes/no)	11/41	8/35	3/6	0.38
*Comorbidities*					
	Diabetes mellitus (type 2/no)	2/50	1/42	1/8	0.32
	Asthma/COPD and using inhalation corticosteroids (yes/no)	5/47	4/39	1/8	1.00
*Most recent glucocorticoid treatment*					
Total daily dose (mg/day), median [IQR]*		25 [20; 30]	25 [20; 30]	30 [20; 37.5]	0.26
Drug (hydrocortisone/cortisone acetate)		35/17	29/14	6/3	0.98
Educated in sick day rules (yes/no)		52/0	43/0	9/0	1.00
				

♦ Educational level was classified using a Dutch education system, comparable to the International Standard Classification of Education (ISCED). This scale ranges from 1 (elementary school not finished) to 7 (university level). *Relative potency cortisone acetate: 0.8.

IQR, interquartile range; SAI, secondary adrenal insufficiency; BMI, body mass index; eGFR, estimated glomerular filtration rate; COPD, chronic obstructive pulmonary disease.

### 3.2 Adrenal Crisis

There were 9 (17%) patients with in total 11 (range 1-3) adrenal crisis. This corresponds to 1.7 crisis per 100 patient-years at risk. All the patients were admitted to the regular ward. The average duration of the hospital stay was 2 days with maximum duration of 7 days. All patients had normal electrolytes during admission and were hemodynamically stable (**Table 2**). 5 out of 11 patients had doubled their hydrocortisone dosage during illness. None of the patients had tripled their hydrocortisone. In 4 out of 11 cases, patients had received an intramuscular emergency administration of glucocorticoids at home. The cause of the AC was an infection in 9 out of 11 of the crises.

**Table 2:** Adrenal crisis details.

		Adrenal crisis (n = 11)
Number of crisis per patient, median [min, max]		1 [1;3]
Glucocorticoid dose adjustment at home		
Double dose (yes/no)		5/6
Triple dose (yes/no)		0/11
Emergency glucocorticoid administration i.m. given at home? (yes/no)		4/7
Type of hospital admission (regular ward/intensive care)		11/0
Duration of hospital stay (days), median [min, max]		2 [1; 7]
*Precipitating factors*		
Infection		9
	Airway/gastrointestinal/other	1/2/4
	Positive culture PCR (yes/no)	4/5
Syncope		1
Medication non-adherence		1
*Findings on admission*		
Systolic blood pressure (mmHg), median [IQR]		129 [119; 131]
Diastolic blood pressure (mmHg), median [IQR]		76 [70; 81]
Sodium (mmol/L), median [IQR]		142 [140; 143]
Potassium (mmol/L), median [IQR]		4.1 [3.6; 4.3]

BPM, beats per minute; IQR, interquartile range; PCR, polymerase chain reaction.

At the lower hydrocortisone dose (0.2-0.3 mg/kg per day)

### 3.3 Plasma and Urinary Concentrations of Steroids and Pharmacokinetics

#### 3.3.1 Plasma Cortisol, Cortisone and CBG

Plasma levels of cortisol and cortisone one hour and five hours after ingestion of hydrocortisone did not differ between the AC + group and the AC- group (**Table 3**). Cortisol binding globulin (CBG) levels were also not different between groups.

**Table 3:** Pharmacokinetic parameters of free cortisol and total cortisol as well as plasma concentrations of cortisol, cortisone and CBG on the lower dose hydrocortisone (0.2-0.3 mg/kg/day) in patients with or without a history of an adrenal crisis.

		Adrenal crisis - (n = 37)	Adrenal crisis + (n = 8)	P value
*Total cortisol*				
	CL (L/H)	11.7 [8.0; 17.4]	12.6 [8.4; 17.1]	0.69
	V_d_ (L)	33.7 [26.0; 46.6]	29.8 [22.2; 50.1]	0.76
	T _½_ (h)	1.82 [1.43; 3.05]	1.68 [1.30; 2.47]	0.33
	AUC_24h_ (h*nmol/L)	3956.1 [2791.9; 5364.0]	3539.8 [2969.9; 5066.4]	0.61
*Free cortisol*				
	CL (L/H)	220.6 [168.2; 334.9]	245.0 [157.1; 364.4]	0.87
	V_d_ (L)	422.3 [315.9; 627.3]	439.6 [234.7; 746.5]	0.90
	T _½_ (h)	1.20 [1.00; 1.65]	1.42 [0.83; 1.87]	0.78
	AUC_24h_ (h*nmol/L)	217.2 [144.7; 260.2]	188.9 [132.0; 267.9]	0.85
				
Cortisol level 1h after HC ingestion		498.6 [387.4; 597.8]	516 [341.8; 663.5]	0.73
Cortisone level 1h after HC ingestion		64.8 [52.2; 69.8]	64.4 [55.9; 73.0]	0.90
Cortisol level 5h after HC ingestion		121.7 [76.1; 243.5]	110.9 [68.1; 164.1]	0.46
Cortisone level 5h after HC ingestion		30.6 [21.1; 41.1]	26.9 [19.8; 33.2]	0.51
CBG (ug/mL)		53 [49; 64]	55 [46; 61]	0.68

Data are median [interquartile range].

Cortisol, cortisone levels are nmol/L in plasma.

CL, total body clearance; V_d_, volume of distribution; T_½_, elimination half-life; AUC 24h, 24 hour area under the curve; HC, hydrocortisone; CBG, cortisol binding globulin.

Data are on treatment with the lower dose of hydrocortisone (0.2-0.3mg/kg).

h*nmol/L stand for hours times concentration, the unit of area under the curve. The asterix does not represent anything else.

#### 3.3.2 Pharmacokinetic Parameters

The pharmacokinetic parameters CL, V_d_, T_½_, C_max1_, AUC_1_ and AUC_24h_ of both plasma free cortisol and total cortisol did not significantly differ between the two groups (**Table 3**).

#### 3.3.3 Steroid Precursors of Cortisol and Aldosterone

11-deoxycortisol, corticosterone and 11-deoxycorticosterone did not significantly differ among the two groups (data not shown, subset analysis in 17 versus 4 patients). 24-hour urinary excretion of tetrahydro-11-deoxycortisol (THS) was lower in AC+, albeit not significant (0.1 [0.0; 0.1] vs. 0.0 [0.0; 0.1] µmol/24h, P=0.33)

#### 3.3.4 Urinary Steroid Metabolites

**Figure 1:**
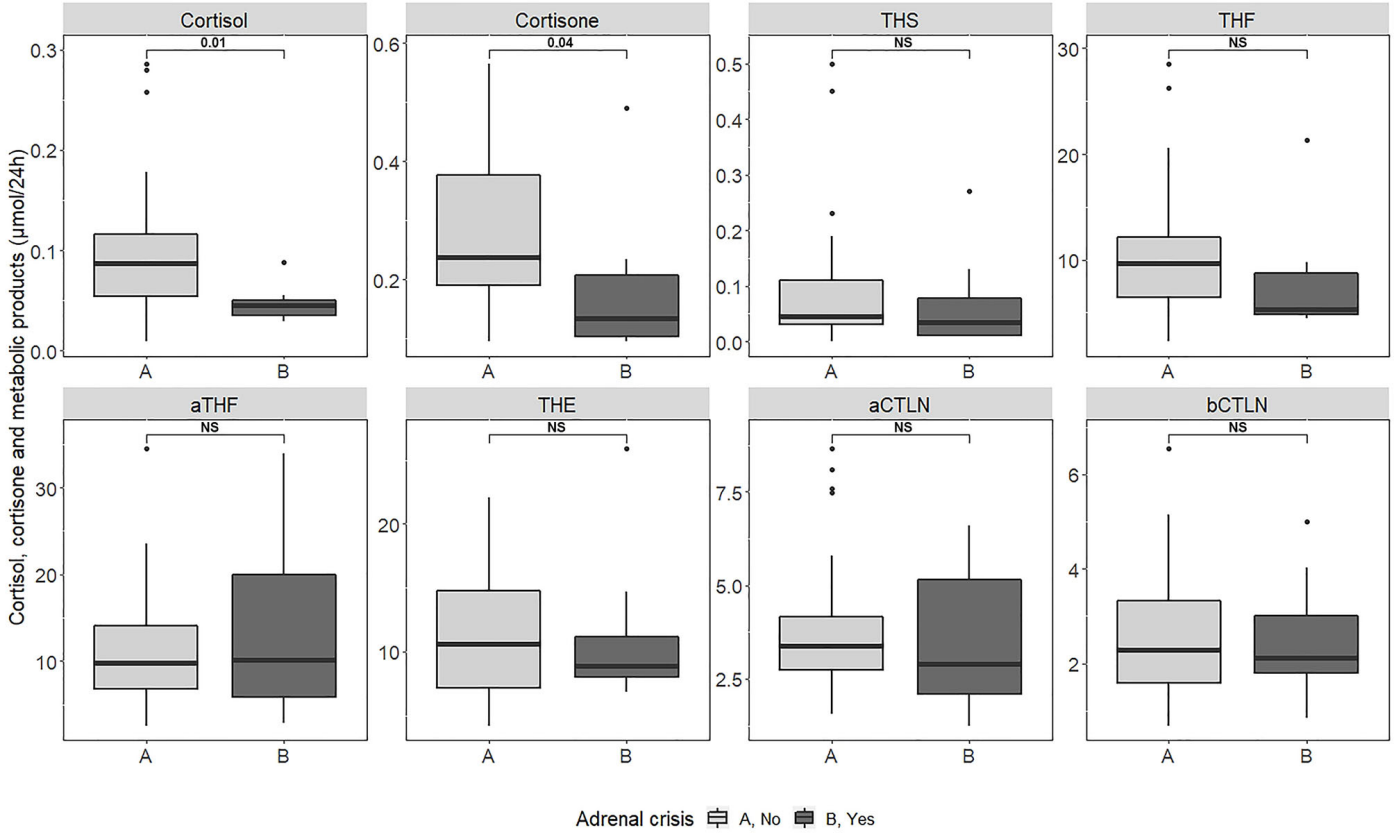
Cortisol and cortisone and metabolic products excretion in 24h urine collections in patients with and without a past history of adrenal crisis. NS, not significant.

The AC+ group had a lower urinary excretion of cortisol and cortisone (0.05 [IQR 0.03; 0.05] vs 0.09[0.05; 0.12] µmol/24h, P =0.01 and 0.13 [0.10; 0.23] vs 0.24 [0.19; 0.38] µmol/24h, P=0.04, **Table 4** and **Figure 1**).

**Table 4:** Urinary steroids and metabolites in patients with and without adrenal crisis on the lower dose hydrocortisone (0.2-0.3 mg/kg/day).

	Adrenal crisis - (n = 43)	Adrenal crisis + (n = 9)	P value
*Total glucocorticoids*	36.66 [25.84; 51.80]	33.61 [22.85; 53.09]	0.76
*Active compounds*			
Cortisol	0.09 [0.05; 0.12]	0.05 [0.03; 0.05]	0.01
Cortisone	0.24 [0.19; 0.38]	0.13 [0.10; 0.23]	0.04
*Metabolites*			
THS	0.05 [0.03; 0.11]	0.03 [0.01; 0.11]	0.33
THE	10.64 [7.10; 14.97]	8.92 [7.52; 13.53]	0.80
THF	9.72 [6.36; 12.33]	5.28 [4.83; 9.44]	0.09
allo-THF	9.86 [6.55; 14.09]	10.21 [5.73; 24.28]	0.76
α-CTLN	3.37 [2.73; 4.35]	2.89 [1.85; 5.38]	0.54
β-CTLN	2.28 [1.54; 3.38]	2.11 [1.80; 3.69]	0.97
*Enzymes*			
11βHSD type 1	1.90 [1.68; 2.29]	1.60 [1.33; 2.52]	0.85
11βHSD type 2	0.33 [0.24; 0.44]	0.33 [0.16; 0.52]	0.85
5α-reductase	0.89 [0.75; 1.22]	0.87 [0.41; 0.94]	0.44

Data are median [Interquartile range]. Units are µmol/24h.

THS, tetrahydro-11-deoxycortisol; THE, tetrahydrocortisone; THA, tetrahydro-11-dehydrocorticosterone; THB, tetrahydrocorticosterone; 5α-THB, 5α tetrahydrocorticosterone; THF, tetrahydrocortisol; allo-THF, allo-tetrahydrocortisol; α-CTLN, α-cortolone; β-CTLN, β-cortolone.

11βHSD type 1; (THF + allo-THF)/THE, 11βHSD type 2; Cortisol/cortisone; Total glucocorticoid metabolite excretion, THF + allo-THF + THE + α-CTLN + β-CTLN; 5α-reductase: allo-THF/THF.

Urinary excretion of metabolites of cortisol and cortisone did not differ significantly among the two groups (**Table 4** and **Figure 1**).

### 3.4 Glucocorticoid Sensitive Pathways

#### 3.4.1 The Tryptophan-Kynurenine Pathway

Levels of kynurenine in serum were significantly higher in the AC + group (2.64 [2.43; 3.28] vs. 2.23 [1.82; 2.38] µmol/L, P = 0.03). In addition, serum levels of 3-hydroxykynurenine and the ratio kynurenine/tryptophan tended to be higher in the AC+ group (0.04 [0.03; 0.05] vs. 0.05 [0.04; 0.08] µmol/L and 0.03 [0.03; 0.04] vs. 0.04 [0.03; 0.05], both P = 0.06; **Table 5**). None of these analytes were different between the two groups when measured during treatment with the higher hydrocortisone dose (**Table 7**).

**Table 5:** Total serum levels of tryptophan, kynurenine, and 3-hydroxykynurenine, kynurenine to tryptophan ratio, aldosterone, renin and metanephrines in patients on the lower dose hydrocortisone (0.2-0.3 mg/kg/day) with or without a history of an adrenal crisis.

	Adrenal crisis -	Adrenal crisis +	P value
*Tryptophan-kynurenine metabolism*	(n=39)	(n=7)	
Tryptophan (µmol/L)	71.07 [58.75; 83.49]	74.82 [58.53; 80.95]	0.72
Kynurenine (µmol/L)	2.23 [1.82; 2.38]	2.64 [2.43; 3.28]	0.03
3-Hydroxykynurenine (µmol/L)	0.04 [0.03; 0.05]	0.05 [0.04; 0.08]	0.06
Kynurenine/tryptophan ratio	0.03 [0.03; 0.04]	0.04 [0.03; 0.05]	0.06
*Renin-angiotensin-aldosterone system*	(n=38)	(n=6)	
Aldosterone (pmol/L)	135.00 [67.00; 263.00]	200.50 [159.25; 330.25]	0.16
Renin (pg/mL)	10.75 [6.56; 17.73]	16.40 [11.95; 44.60]	0.09
Aldosterone/renin ratio (pmol/ng)	12.20 [3.24; 21.25]	12.26 [7.24; 17.43]	0.98
Use of RAAS interfering medication (yes/no)	13/30	4/5	0.45
Potassium (mmol/L)	3.90 [3.65; 4.00]	3.90 [3.75; 4.15]	0.44
*Adrenal medulla activity*	(n=39)	(n=6)	
Normetanephrine (nmol/L)	0.61 [0.49; 0.84]	0.59 [0.44; 0.69]	0.46
Metanephrine (nmol/L)	0.13 [0.10; 0.20]	0.16 [0.09; 0.20]	0.79
3-Methoxytyramine (nmol/L)	0.03 [0.02; 0.04]	0.03 [0.02; 0.04]	0.71

Data are median [interquartile range].

Data are on treatment with the lower dose of hydrocortisone (0.2-0.3 mg/kg/day).

RAAS interfering medication: angiotensin-converting enzyme inhibitors, angiotensin receptor blockers, betablockers.

RAAS, renin angiotensin aldosterone system.

#### 3.4.2 Plasma Renin and Aldosterone

There was a trend towards significant for differences in plasma levels of renin in the AC + group (10.75 [6.56; 17.73] pg/mL vs. 16.40 [11.95; 44.60] pg/mL, P=0,09; **Table 5**). Levels of aldosterone did not differ significantly between the AC+ group and the AC- group.

#### 3.4.3 Plasma Metanephrines

Levels of normetanephrine, metanephrine and 3-methoxytyramine did not significantly differ among the two groups (**Table 5**).

#### 3.4.4 Quality of Life Questionnaires

Patients in the AC + group reported significantly more general fatigue (Z-score -0.16 [-0.80; 0.28] vs. 1.02 [-0.11; 1.42], P = 0.04). Levels of physical functioning did not differ among the two groups. There was a trend for more pain and anxiety in the AC + group (Z-score -0.39 [-0.74; 0.05] vs -0.01 [-0.45; 0.91], P = 0.08; Z score -0.58 [-1.14; -0.49] vs. 0.11 [-0.57; 0.74], P = 0.06, **Table 6**).

**Table 6:** Perceived pain, fatigue, physical functioning and mood in patient with and without adrenal crisis on the lower dose hydrocortisone (0.2-0.3 mg/kg/day).

	Adrenal crisis - (n = 38)	Adrenal crisis + (n = 8)	P value
*PHQ-9*			
Pain	-0.39 [-0.74; 0.05]	-0.01 [-0.45; 0,91]	0.08
*MFI-20*			
General Fatigue	-0.16 [-0.80; 0.28]	1.02 [-0.11; 1.42]	0.04
*RAND-36*			
Physical functioning	0.42 [-0.12; 0.53]	0.20 [-0.07; 0.31]	0.23
*HADS*			
Anxiety	-0.58 [-1.14; -0.19]	0.11 [-0.57; 0.74]	0.06
Depression	-0.12 [-0.73; 0.79]	0.18 [-0.66; 1.62]	0.66

Data are represented in Z-scores.

Data are median [interquartile range]. PHQ-9, Physical Health Questionnaire 9; MFI20, multidimensional Fatigue Index-20; RAND-36, 36-Item Short Form Health Survey; HADS, Hospital Anxiety and Depression Scale.

### 3.5 Glucocorticoid Sensitivity-Altering Glucocorticoid Receptor Polymorphisms

There was no association between the GR polymorphisms (typed as GR hypersensitive, resistant or wild-type variants) and the occurrence of an AC (data not shown).

### 3.6 At the Higher Hydrocortisone Dose (0.4-0.6 mg/kg per Day) for Variables of Interest With P<0.1 at the Lower Hydrocortisone Dose

Serum concentrations of kynurenine did not differ between the two groups at the higher dose of hydrocortisone. Aldosterone was significantly higher in the AC+ group (91.5 [<0.4; 218.5] vs. 249.0 [115.0; 337.0] pmol/L, P = 0.04), but renin was not. Urinary cortisol and cortisone remained lower in the AC+ group (0.31 [0.23; 0.43] vs. 0.18 [0.11; 0.26] µmol/24h, P = 0.01 and 0.48 [0.38; 0.71] vs. 0.32 [0.22; 0.38] µmol/24h, <0.01).

Perceived pain, general fatigue and anxiety did not differ between patients with or without an AC (**Table 7**).

**Table 7:** Urinary glucocorticoid excretion, tryptophan-kynurenine metabolism, renin-angiotensin-aldosterone system and self-reported aspects of quality in life in patients on the higher dose hydrocortisone (0.4-0.6 mg/kg/day).

	Adrenal crisis -	Adrenal crisis +	P value
*Urinary glucocorticoid excretion*	(n=38)	(n=7)	
24h Urinary cortisol (µmol/24h)	0.31 [0.23; 0.43]	0.18 [0.11; 0.26]	0.01
24h Urinary cortisone (µmol/24h)	0.48 [0.38; 0.71]	0.32 [0.22; 0.38]	<0.01
THS (µmol/24h)	0.05 [0.03; 0.08]	0.05 [0.02; 0.09]	0.79
THF (µmol/24h)	21.91 [15.70; 25.56]	21.60 [15.37; 27.42]	0.87
*Tryptophan-kynurenine metabolism*	(n=38)	(n=7)	
Serum kynurenine (umol/L)	1.89 [1.52; 2.45]	2.05 [1.53; 3.18]	0.57
Serum 3-hydroxykynurenine (umol/L)	0.03 [0.02; 0.05]	0.04 [0.04; 0.07]	0.16
Serum kynurenine/tryptophan ratio	0.03 [0.02; 0.03]	0.02 [0.02; 0.03]	0.59
*Renin-angiotensin-aldosterone system*	(n=38)	(n=7)	
Serum aldosterone (pmol/L)	91.5 [<0.4; 218.5]	249.0 [115.0; 337.0]	0.04
Plasma renin (pg/mL)	8.63 [5.89; 14.78]	22.55 [5.93; 38.72]	0.23
Potassium (mmol/L)	3.70 [3.60; 3.90]	3.90 [3.75; 4.10]	0.03
*Reported symptoms*	(n=38)	(n=8)	
Pain, z-score	-0.41 [-0.71; 0.12]	-0.24 [-0.55; 0.58]	0.32
General fatigue, z-score	0.01 [-0.75; 0.98]	-0.66 [-1.14; 0.26]	0.17
Anxiety, z-score	-0.53 [-1.14; -0.03]	-0.02 [-0.58; 1.08]	0.18

Data are represented in Z-scores. Data are median [interquartile range].

Variables of interest were selected based on P<0.1 (+ aldosterone) on lower dose hydrocortisone treatment.

THS, tetrahydro-11-deoxycortisol; THF, tetrahydrocortisol.

## 4 Discussion

To the best of our knowledge, this is the first study to investigate a biological predisposition for the risk of an adrenal crisis by means of extensive examination of cortisol pharmacokinetics in combination with several glucocorticoid sensitive pathways in a well-defined cohort of patients with secondary adrenal insufficiency. Patients with a history of an adrenal crisis could be distinguished from those with a negative history by a lower urinary excretion of cortisol and cortisone and alterations in glucocorticoid sensitive pathways like the tryptophan-kynurenine pathway and the level of general fatigue. Collectively, the emerging picture is compatible with a reduced efficacy of hydrocortisone substitution treatment at the usually recommended lower maintenance doses in patients at risk for developing an adrenal crisis.

The AC+ group demonstrated lower urinary cortisol and cortisone excretion compared to the AC-group at both the lower and higher hydrocortisone substitution dose. We did not find differences in pharmacokinetic parameters such as clearance, volume of distribution or elimination half‐life of cortisol between these groups. In addition, the urinary excretion profile of metabolites of cortisol and cortisone and their precursors were also comparable in either group. We speculate that the renal excretion of cortisol between the AC+ group and the AC- group possibly explains the differences, but further research should be done to confirm this. The precise mechanism of our findings remains therefore elusive, as we did not find any differences in either the estimated glomerular filtration rate or the enzymatic activity of 11β-hydroxysteroid dehydrogenase type 2.

The pharmacodynamic part of our study showed that the serum kynurenine concentration was significantly higher in the AC+ compared to the AC- group. The first step of the kynurenine pathway is conversion of tryptophan in N′-formylkynurenine, which consecutively becomes hydrolyzed into kynurenine (25). This process involves two enzymes, i.e. tryptophan 2,3-dioxygenase (TDO) and indoleamine 2,3-dioxygenase (IDO). IDO is activated by pro-inflammatory cytokines and reactive oxygen species (ROS). Induction of TDO by activation of the glucocorticoid receptor has been shown in several studies (25–29). These studies were predominantly performed in animals and examined the effect of a single dose of very potent glucocorticoids. However, in prior research we demonstrated an inverse relationship between hydrocortisone dose and serum kynurenine concentration after a treatment period of 10 weeks in study patients of the current RCT (21). Therefore, serum kynurenine could potentially serve as an index of glucocorticoid sensitivity. The elevated level of serum kynurenine in the AC+-group somehow suggests a reduced hydrocortisone efficacy in these patients, which cannot be ascribed to differences in plasma cortisol levels. However, the responsible pharmacodynamic mechanism underlying this lower cortisol sensitivity in the AC+ group remains speculative. On the higher hydrocortisone dose, neither serum kynurenine concentration nor quality of life variables were different between the AC+ and AC- group, suggesting the AC+ group requires a higher substitution dose to compensate for the apparent lower cortisol sensitivity.

Alternative hypotheses to explain differences in the occurrence of adrenal crisis are insufficient education on sick-day rules including self-administration of intramuscular hydrocortisone injections or residual endogenous production of cortisol. Regarding patient education, it should be noted that participants in this study all had received extensive and uniform instructions on sick-day rules during the time of the randomized controlled trial. In addition, during this 20-week study both the AC+ and AC- group did not differ in application of doubling or tripling of the hydrocortisone dose (data not shown), emphasizing that our groups were comparable at this point. Furthermore, with regard to residual cortisol production, we did not find differences in plasma concentration of 11-deoxy-cortisol and urinary THS, although these data must be interpreted with caution as this measurement was not available in all study subjects (30).

We also included aldosterone and renin in our analysis as markers of secondary activation of the RAAS as this was previously shown to be responsive to the hydrocortisone dose and may reflect glucocorticoid mediated mineralocorticoid receptor (MR) activation (19). We found in the AC+ group a trend for higher renin activity at the lower dose, and significantly higher aldosterone at the higher dose. These results suggest increased RAAS activation in AC+ group, secondary to reduced hydrocortisone mediated MR activation at the same dose as the AC- group. However, this should be interpreted with caution as blood pressure between groups was similar and group size was small.

On the higher dose, urinary cortisol and cortisone excretion remained lower in the AC+ group. However, significant differences in the kynurenine pathway and associated quality of life parameters disappeared on the higher dosage of HC. A possible explanation for this phenomenon could be, that the group who has proved to be vulnerable for an AC is actually undersubstituted. This group might need a higher hydrocortisone dosage, which is not reflected in lower plasma cortisol concentrations in serum or pharmacokinetic parameters but resonates in glucocorticoid sensitive pathways as an expression of shortage of hydrocortisone at the receptor level.

The strength of this study is the high-quality data within the framework of a randomized controlled trial. This trial provides extensive phenotyping of a homogenous group of SAI patients on a standardized dose of hydrocortisone. In addition, we analysed the urine samples using a GC-MS/MS method, which provides higher analytical specificity compared to the previous GC/MS method (31). Furthermore, we used a state-of-the-art LC-MS/MS method to measure analytes in the kynurenine pathway (20).

A few limitations need to be addressed. First, as stated above, this study is hypothesis generating. Further studies should be specifically designed to address our research question. Secondly, as a consequence of retrospective data retrieval on AC, there were some missing data concerning hospital admission. Furthermore, due to the retrospective study design, for some analysis, there was not enough biomaterial available. Therefore not all measurements could be performed in every study participant. In addition, we did not include all cortisol metabolites (e.g. α-cortol) into our analysis. Moreover, our study encompassed only 9 individuals with a past history of one or more AC. This relatively small number not only limited the statistical power but might also have increased the risk of ‘false positive’ findings, as a result of coincidental outliers. It should be noted, however, that all glucocorticoid variables were assessed under the strictly defined conditions of a randomized controlled trial. In addition, our findings also fit into a plausible pathophysiological model of potentially reduced glucocorticoid bioavailability and sensitivity in patients prone to develop AC.

In conclusion, this study suggests that patients at risk for an adrenal crisis demonstrate differences in urinary excretion of cortisol and cortisone, as well as in glucocorticoid sensitivity, hinting to a biological predisposition for an adrenal crisis in these patients. Prospective studies are needed to elucidate whether these characteristics will identify patients at high risk for an adrenal crisis and if this risk can be reduced successfully by interventions directed at normalization of these alterations.

## Data Availability Statement

The original contributions presented in the study are included in the article/supplementary material. Further inquiries can be directed to the corresponding author.

## Ethics Statement

The studies involving human participants were reviewed and approved by METc Groningen. The patients/participants provided their written informed consent to participate in this study.

## Author Contributions

Participated in research design: AB and AV. Participated in the writing of the paper: AV, AB, MF, and MK. Participated in the performance of the research: AV, AB, MF, and MK. Contributed new reagents or analytic tools: MF. Participated in data analysis: AV, AB, MF, and MK. All authors contributed to the article and approved the submitted version.

## Conflict of Interest

The authors declare that the research was conducted in the absence of any commercial or financial relationships that could be construed as a potential conflict of interest.

## Publisher’s Note

All claims expressed in this article are solely those of the authors and do not necessarily represent those of their affiliated organizations, or those of the publisher, the editors and the reviewers. Any product that may be evaluated in this article, or claim that may be made by its manufacturer, is not guaranteed or endorsed by the publisher.
